# Spatial exploration of the CDC’s Social Vulnerability Index and heat-related health outcomes in Georgia

**DOI:** 10.1016/j.ijdrr.2020.101517

**Published:** 2020-06

**Authors:** Erica Adams Lehnert, Grete Wilt, Barry Flanagan, Elaine Hallisey

**Affiliations:** Geospatial Research, Analysis and Services Program, Division of Toxicology and Human Health Sciences, ATSDR, 4770 Buford Hwy NE, Mailstop S106-5, Atlanta, GA, 30341-3717, USA

**Keywords:** Social vulnerability, Heat-related health outcomes, Spatial analysis

## Abstract

Heat-related illness, an environmental exposure-related outcome commonly treated in U.S. hospital emergency departments (ED), is likely to rise with increased incidence of heat events related to climate change. Few studies demonstrate the spatial and statistical relationship of social vulnerability and heat-related health outcomes.

We explore relationships of Georgia county-level heat-related ED visits and mortality rates (2002–2008), with CDC’s Social Vulnerability Index (CDC SVI). Bivariate Moran’s I analysis revealed significant clustering of high SVI rank and high heat-related ED visit rates (0.211, p < 0.001) and high smoothed mortality rates (0.210, *p* < 0.001). Regression revealed that for each 10% increase in SVI ranking, ED visit rates significantly increased by a factor of 1.18 (95% CI = 1.17–1.19), and mortality rates significantly increased by a factor of 1.31 (95% CI = 1.16–1.47).

CDC SVI values are spatially linked and significantly associated with heat-related ED visit, and mortality rates in Georgia.

## 1. Introduction

Extreme heat-related illness (defined by ICD-9992.0–992.7 or E–900.0) is one of the most common environmental exposure-related illnesses treated in U.S. hospital emergency departments [1,2]. Deaths vary nationally between 600 and 1800 annually [3], accounting for a substantial percentage of all weather-related fatalities in the United States [4]. Extreme heat presents in the human body with loss of control of internal temperature, and manifests in symptoms including heat cramps, heat exhaustion and heatstroke, as well as co-morbidities involving the nervous, respiratory, cardiovascular, genito-urinary systems, and diabetes-related conditions [5–7].

Previous studies have examined potential predictors of hospitalizations and mortality from extreme heat events. Rosenthal et al. [8] suggested that heat morbidity in individuals over 65 years of age is driven by poverty, poor housing conditions, lower rates of air conditioning, and impervious land cover at a neighborhood scale. Both older age and living alone have been shown to be strong predictors of heat stress in some regions of the United States [9–11]. Pillai et al. [1] found that being male, elderly, or having middle-lower socioeconomic status are predictors for being admitted to a hospital for heat illness. Wilhelmi et al. [12] reported that social determinants play an important role in extreme heat-related incidents in major cities. Maier et al. [13] found that social isolation and elderly populations were the strongest predictors in rural regions, whereas poverty and non-white status were the strongest indicators in urban regions. Trends in demographics point to a changing population structure that suggests an increase in heat morbidity and mortality globally in the more vulnerable older population (≥60 years), which is projected to triple from 2000 to 2050 [14,15]. Further, expected migration to cities has the potential to put more individuals at risk from the urban heat island effect [16]. Wilson et al. [17] created a heat vulnerability index that identified the highest scoring heat vulnerable areas to be in counties in the South and in metropolitan areas in the Northeast and Midwest regions of the United States.

Many of the individual predictors cited above represent socio-demographic population characteristics common to cities and counties of the American South. Pillai et al. [1] provide extensive descriptive analysis of heat-related illness in Georgia. In brief, heat-related illness accounted for 13,784 emergency department (ED) visits from 2002 to 2008, with approximately 7% of those resulting in hospital admissions [1]. Residents of Georgia are especially vulnerable to heat stress, as it is a southern state with a humid, subtropical climate, large rural areas of poverty, a significant non-white population, and the primary urban agglomeration of Atlanta. In addition to previously cited predictive factors, risk of heat stress is increased for older males and increases on a gradient moving south and towards the coast [1].

While existing literature examines individual predictors, there are few previous studies of the relationship between a composite measure of overall social vulnerability and extreme heat-related ED visits and mortality. Social vulnerability defines the extent to which a community exhibits certain social characteristics that may resilience [18], and may be influenced by multiple factors such as employment, income, education [19,20], housing type, age, race and ethnicity [19,21]. Any of these and other social characteristics may affect that community’s ability to prevent human suffering and financial loss when hazards threaten. Evidence suggests that socially vulnerable populations are often less prepared for potential disasters and more likely to experience adverse effects during a disaster [19,22,23]. Given the need to include many factors, indices have become a common tool to measure vulnerability [24] for use in hazard preparedness and planning. While several national measures of social vulnerability exist [19,22], Reid et al. [7] developed a more specific Heat Vulnerability Index for major metropolitan areas across the United States using risk factors, including population with diabetes; households below poverty line; race other than white; living alone; age > 65 years and living alone; less than a high school diploma; no green space; no central air conditioning; and no air conditioning of any kind. This index was created at the broad scale of metropolitan area and did not isolate social vulnerability as a predictor for heat-related health outcomes [25]. Additionally, Chuang et al.(2015) evaluated Reid’s index and found that the heat vulnerability index was too general to be useful in localized contexts, such as cities [26]. Johnson et al. [27] built a heat index combining environmental and social indicators that explained 80% of deaths in highest risk zones of cities. A need exists for finer scale studies across all metropolitan and non-metropolitan areas especially in climatologically vulnerable areas such as the Southern United States.

In this study, statistical and spatial analyses were used to examine the utility of a composite measure of social vulnerability on heat-related ED visits, and mortality at the county level in Georgia. The primary objective was to determine the utility of a composite measure of social vulnerability as a standalone tool in identifying counties that are more likely to experience heat-related health outcomes and thus may require increased resources and communications during a hazardous heat event.

## 2. Methods

### 2.1. Data

De-identified, patient-level data for heat-related ED visits and mortality were obtained from Georgia Department of Public Health (GA DPH), Office of Health Indicators for Planning (OHIP) [28], based on their International Classification of Diseases (ICD) codes, for all 159 counties in Georgia. Table 1 shows a descriptive breakdown of ED visits and mortality across counties in Georgia.

**Fig. 1.**
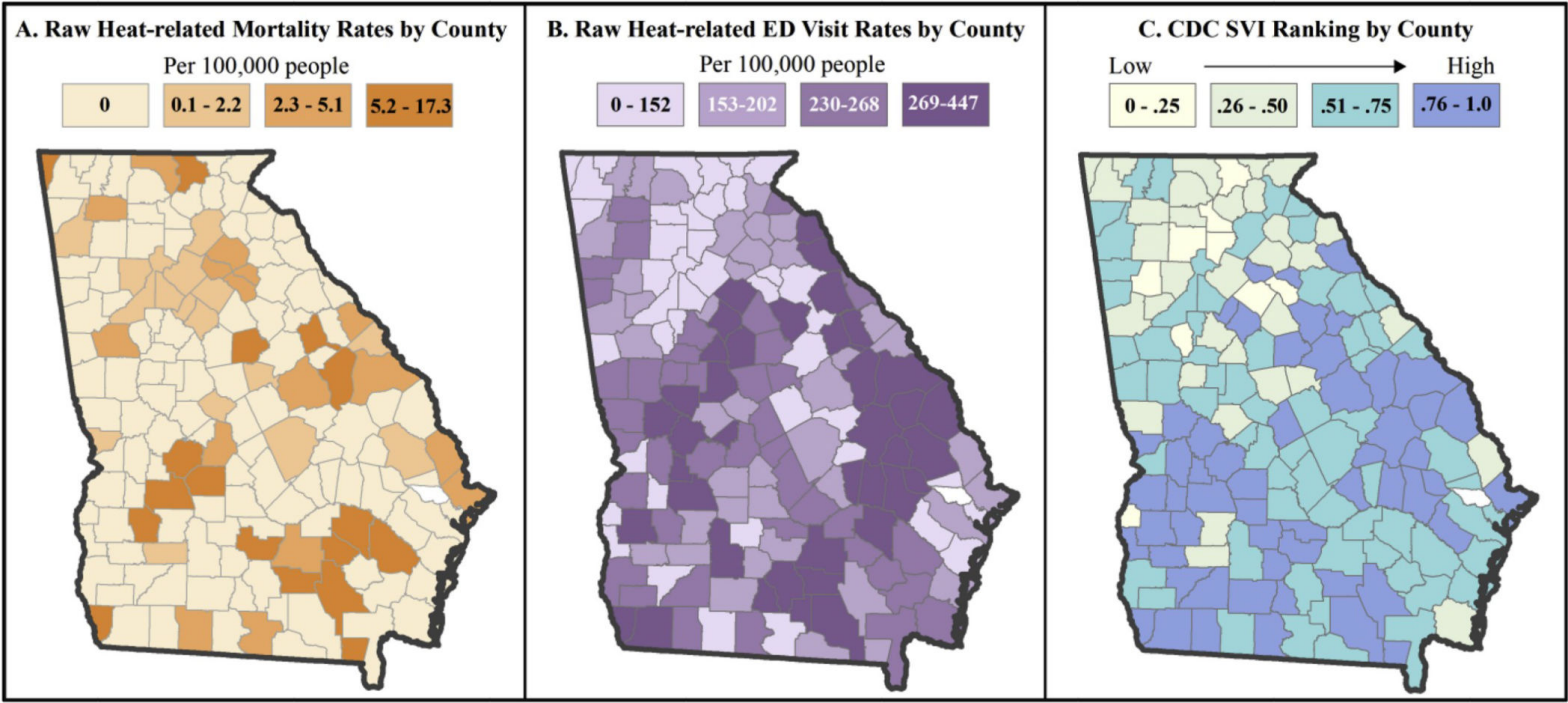
Distribution Maps of Heat-Related Mortality Raw Rates, ED Visit Rates, SVI (2002–2008). The first two maps provide spatial context for heat-related mortality and ED visit rates across Georgia, while the third map provides spatial context for social vulnerability across Georgia. Visual inspection of these maps generally reveals higher rates in areas of higher social vulnerability, presenting evidence for further spatial and statistical exploration. All maps are visualized using quartile class breaks.

The following criteria were required for an entry to be used in the study: The death or ED visit took place in May through September of 2002–2008 and at least one of the top ten ICD entries for cause of death or illness was coded as ICD-9992.0–992.7 or E–900.0 (ICD-10 code equivalent was used for deaths). These ICD codes represent heat-caused stroke, syncope, cramps, exhaustion, fatigue, edema, and accidents caused by excessive heat. County of residence was included in the dataset and used as the geographic unit of analysis and all 159 Georgia counties were included in all analyses. The ED visit data contained a binary field noting whether the ED visit resulted in hospital admission or release, which we used to explore the relationship between the Centers for Disease Control and Prevention Social Vulnerability Index (CDC SVI) and heat-related hospital admissions from the ED. Fig. 1A and B displays raw mortality and ED visit rates from the study period across Georgia counties.

CDC SVI was retrieved from the publicly available website (svi.cdc.gov). This analysis uses county level CDC SVI data for the state of Georgia from the year 2000 (Fig. 1C). The development of CDC SVI is described in detail in Flanagan et al. [22]. Briefly, the 2000 version of CDC SVI is derived from summed percentile rankings at census tract level of fifteen U.S. Census variables, shown in Table 2. These variables were selected based on extensive review of the literature [19,20,23,29,30], and represent socioeconomic and demographic characteristics within four themes: socioeconomic status, household composition, minority status and language, and housing and transportation. CDC SVI scores range from 0 to 1, with 1 representing the highest level of social vulnerability [22]. The original CDC SVI dataset provides rankings at tract level for each variable, each theme, and an overall ranking.

Each of the four themes and the composite overall social vulnerability score were evaluated at county-level as potential indicators of vulnerability. Although data were de-identified, Georgia Department of Public Health linked and provided SVI values, rounded to the nearest tenth, for each patient’s census tract of residence. Because census tract rankings are a more precise measure of social vulnerability than county-level rankings, the provided values were averaged for patients by county to provide a single CDC SVI score representing patients within each county. Census-tract level CDC SVI rankings did not accompany the mortality data, thus county CDC SVI rankings were used for spatial analysis.

### 2.2. Data analysis

#### 2.2.1. Spatial autocorrelation analysis

We conducted univariate and bivariate tests for spatial autocorrelation using both global and local Moran’s I to examine the strength and significance of spatial effects. Spatial autocorrelation is a measure of similarity (positive) or dissimilarity (negative) of a variable with itself or other variables in space. Positive spatial autocorrelation (>0) signifies clustering of similar values, whereas negative spatial autocorrelation (<0) indicates dissimilar values are adjacent to one another. An output value of 0 signifies complete spatial randomness. The farther the output value is from 0, the stronger the spatial autocorrelation.

Univariate global and local Moran’s I tests were conducted for ED visits, heat mortality, and CDC SVI percentile rankings individually. Bivariate global and local Moran’s I tests were conducted for both ED visits and heat mortality with overall SVI percentile ranking score and again with each of the four CDC SVI themes. In this study, a low number of deaths (<1 per 100,000) in 70% of the 159 counties in Georgia (see Fig. 1) rendered mortality rates spatially unstable. Empirical Bayesian Smoothing (EBS) was applied to stabilize mortality rates and univariate and bivariate tests for spatial autocorrelation were repeated. EBS uses the mean number of deaths and population of a county and its first-order neighbors to assign a new expected rate. The more unstable a rate is, the more information the algorithm will draw from the neighboring counties to smooth the rate. This method is commonly used to stabilize rates in areas with small numbers, as is the case with mortality in this study [31].

All Moran’s I analyses were conducted using GeoDa Software (GeoDa 1.6.7). Both univariate and bivariate Moran’s I tests were run using 999 Markov Chain repetitions and a first order queen’s contiguity spatial weights matrix. This method uses the values from all first-order neighboring counties to determine if the area has a higher or lower mean, from which the degree of spatial autocorrelation is assessed.

Additionally, we conducted geographically weighted Pearson correlation between both heat mortality and morbidity, respectively, and overall CDC SVI score using the GWmodel package for geographically weighted summary statistics package in R3.2. This analysis examines whether the association between two variables varies geographically, using a moving window approach, and can be used to validate findings from local Moran’s I tests.

#### 2.2.2. Regression analysis

Due to the spatial nature of heat-related data, we explored OLS models of heat-related ED visits rates and mortality rates for any violations in uncorrelated error and independent observations. Heat-related mortality rates were log-transformed to fit a normal distribution. We defined a spatial weights matrix of first order queen’s contiguity in GeoDa. The OLS regression diagnostics, including the Lagrange Multiplier (LM) for spatial lag and Moran’s I residual autocorrelation were conducted for all regressions. We concluded regressions with heat-related ED visit rates should be run using spatial lag models due to significant LM terms in the OLS models. OLS regression indicated no significant spatial lag terms for heat-related mortality rates. We selected a Poisson model for mortality regressions as this distribution is tailored to work with datasets containing low numbers and zeros. For all regressions each of the four CDC SVI themes (Table 1) and the overall CDC SVI percentile rank per county were examined for potential associations with heat-related ED visit rates and heat-related mortality.

## 3. Results

### 3.1. Descriptive statistics

In Georgia, from years 2002 through 2008, there were 14,244 heat-related ED visits, of which 1034 (7.26%) resulted in hospitalization, as well as 94 heat-related mortalities. Table 3 compares descriptive statistics, including race, sex, and age of the Georgia population with those of the population who had ED visits due to heat exposure and the population who died of heat-related causes.

While the majority of the state population identifies as white, ED visits were disproportionately higher among blacks and represented over half of all mortality. Similarly, males had much higher ED visit and mortality rates than females, though females number slightly over half of the population. The 17 and under age group, which makes up 26.47% of the GA population represents only 17.07% of ED visits and 14.89% of heat-related mortality, while the 18–39 age group, which makes up 31.86% of the GA population represents 40.59% of ED visits. The 65 and older age group, comprising but 9.58% of the GA population, represents 36.17% of mortality. As expected, mortality rates, as a percentage of total mortality, were disproportionately and progressively higher for the two older population groups, despite being lower percentages of the overall population.

### 3.2. Spatial autocorrelation analysis

#### 3.2.1. Univariate

Results of the univariate global Moran’s I revealed significant positive spatial autocorrelation in overall CDC SVI rankings (0.43, *p* < 0.001) and heat-related ED visit rates (0.32, *p* < 0.001). No significance was observed in the Local Moran’s I tests for heat-related mortality rates. However, there were significant clusters observed for heat-related ED visits in southeast Georgia.

#### 3.2.2. Bivariate

**Fig. 2.**
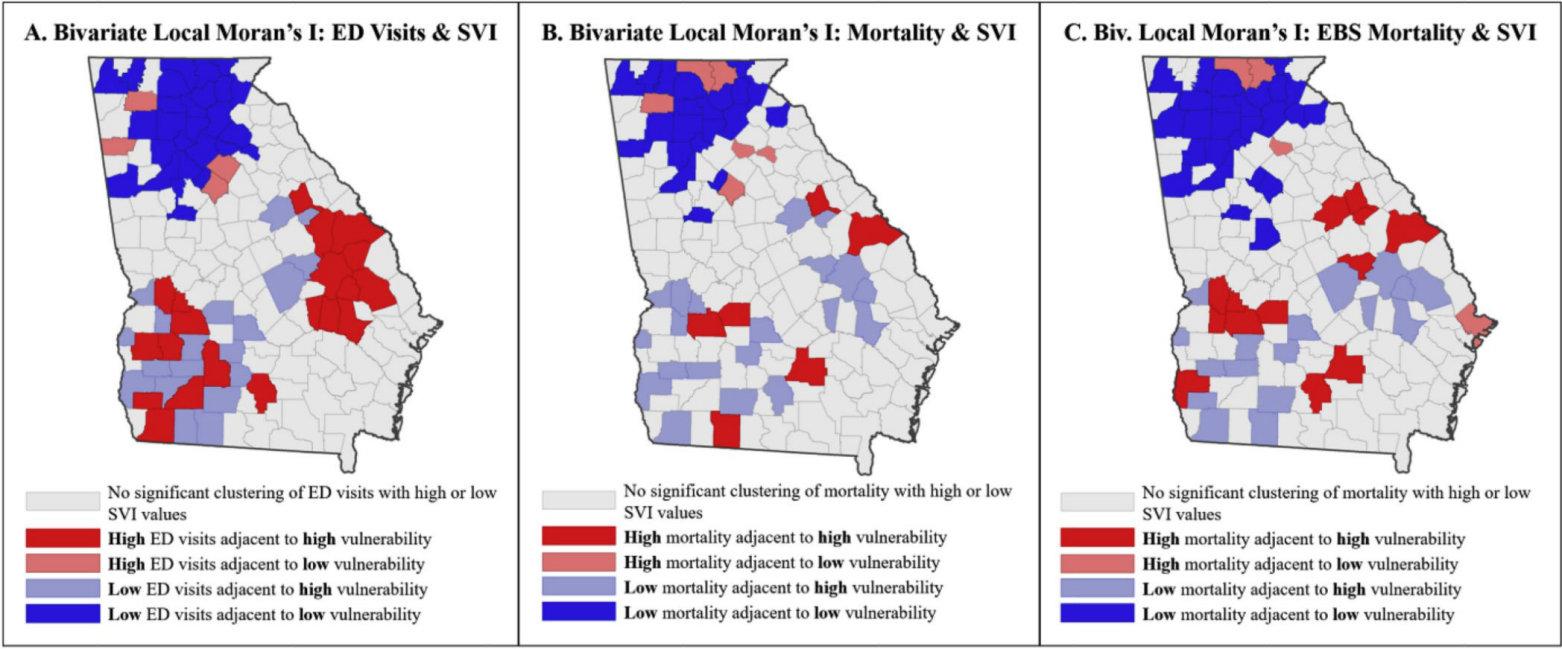
Cluster Maps of Heat-related ED Visit Rates and Mortality Rates with CDC SVI in Georgia, The first map above shows local hot and cold spots of heat-related ED visits and SVI values. The second map shows local hot and cold spots of heat-related mortality and SVI values. The third map show local hot and cold spots of smoothed heat-related mortality and SVI values. (α = 0.05).

Bivariate global Moran’s I analysis of extreme heat-related ED visit rates and overall CDC SVI rank demonstrated clustering of similar values (0.21, *p* < 0.001), meaning globally, areas with high heat-related ED visit rates tended to have high SVI percentile ranks. As expected, given the spatially unstable nature of the mortality rates, the bivariate Moran’s I examining extreme heat-related mortality rates and overall CDC SVI rank showed no significant spatial relationship between mortality rates and SVI ranks. However, the bivariate Moran’s I examining smoothed rates of extreme heat-related mortality and overall SVI rank revealed clustering of similar values (0.21, *p* < 0.001) meaning areas with high heat-related EBS mortality rates had high CDC SVI percentile ranks. Fig. 2 displays the results of the bivariate Local Moran’s I analyses which identifies significant local hot or cold spots.

Breaking down CDC SVI into its four themes (Table 2), the relationships between social vulnerability themes and extreme heat outcomes were further examined (see Table 4). Significant clustering of similar values was found with ED visits and all themes. Significant moderate clustering was found with mortality and socioeconomic status (I = 0.08, p = 0.018), while all other themes displayed spatial randomness. Table 4 shows all bivariate analysis results of mortality and ED visit rates with overall CDC SVI and each CDC SVI theme.

#### 3.2.3. Geographically weighted Pearson’s correlation

There is variability in the ranges for both the geographically weighted Pearson correlation between heat mortality and ED visits and overall CDC SVI by location. For ED visits, the geographically weighted Pearson correlations ranged from −0.71 to 0.75 with a mean of 0.042. For heat mortality, the range was smaller, from −0.38 to 0.43 with a mean of −0.042. The large range of values in these findings is indicative of strong geographic variation in the two relationships. This finding indicates that the relationship between CDC SVI and heat-related ED visits and mortality, respectively, is not consistent across all locations; supporting the variation and number of hot and cold spots identified in the bivariate Local Moran’s I findings seen in Fig. 2.

### 3.3. Regression analysis

#### 3.3.1. Heat-related ED visits and hospitalizations

Spatial lag regression was used to determine the association between SVI and heat-related ED visits. Overall, increases in CDC SVI ranking were significantly associated with increases in number of ED visits by county. For every 10% increase in overall CDC SVI ranking the rate of heat-related ED visits increase by 192.7% (Table 5). All themes had significant positive associations with ED visit rate visits. Theme 2, household composition and disability, with an increase of 205.2%, had the strongest association of all.

#### 3.3.2. Heat-related mortality

As the Moran’s I value was small and the Lagrange Multiplier value in the OLS model was not significant, we proceeded with a non-spatial Poisson regression. For each 10% increase in overall CDC SVI ranking the heat mortality rate increases by a factor of 1.31 (Table 6). Breaking the CDC SVI down into its four component themes shows similar effects, with all themes except minority status and language, Theme 3, significant. For every 10% increase in each individual theme ranking, rates increase by factors of 1.31, 1.31 and 1.40 for socio-economic status, household composition and disability and housing and transportation respectively.

## 4. Discussion

The results of this study are consistent with what is seen in existing literature related to social determinants’ relationship with heat-related health outcomes [1,12]. In addition, this study expands on the spatial linkages between heat-related health outcomes and social determinants of health [7]. Analysis of spatial autocorrelation indicated that social vulnerability, as characterized by CDC SVI, is spatially linked to heat-related ED visits and smoothed mortality rates in Georgia and identified regions that have increased social vulnerability and generally experience increased heat-related health outcomes. These findings suggest that regions of southern Georgia have relatively higher social vulnerability than northern Georgia (Fig. 1C), and pinpoints hot spots of heat-related health outcomes in the southwestern and central eastern regions (Fig. 2A, C). These regions generally coincide with counties included in the South’s historical Black Belt, associated with the use of African-originated enslaved agricultural labor on its plantations [32]. The area remains largely agricultural, where an increased number of individuals performing outdoor labor may be more likely to experience a heat-related health event. Increased education on heat safety may help reduce human vulnerability to heat in Georgia, as well as policy or guidelines restricting time spent in heat without hydration or a break indoors.

The spatial relationship between heat-related health outcomes and the CDC SVI is further substantiated by the significant associations seen in both the spatial lag and Poisson regression analyses. All spatial and statistical tests of individual CDC SVI themes revealed an association with heat-related ED visits and mortality rates. Given these results, it is likely that multiple social determinants are driving the relationship between the social vulnerability measures and the observed heat-related outcomes. This finding displays the utility of a composite social vulnerability indicator as a tool for identifying communities with increased likelihood for heat-related morbidity and mortality in Georgia, as it captures multiple potential contributing factors and affected sub-populations. Further study should be completed to determine if this utility exists in other locations or for health outcomes related to other hazard types. However, in the case of Georgia, it is likely that interventions to minimize heat-related health outcomes will need to consider multiple influencing social factors. For example, education may be helpful to those who lack knowledge related to heat safety but may result in little change if communities and individuals do not have the resources to change their infrastructure or circumstance. Subsidies for air conditioners in lower income areas and for the elderly as well as increased wages and decreased working hours for agricultural workers may be some helpful mitigation strategies from a policy perspective.

The only non-significant term in the mortality regressions and the smallest regression coefficient in the ED visit regressions was Theme 3: minority status and language. The descriptive data in Table 2 shows disproportionately higher numbers of blacks visiting the ED due to heat-related events and dying due to heat-related causes compared to other races. Males had disproportionately high ED visit rates and mortality. The data also suggest that people 65 years of age and older have a disproportionately higher mortality rate, compared to other age groups. Because age-related variables are included in CDC SVI, we did not adjust for age so as to avoid over-explanation in statistical models. Each of these subgroups had varied exposure to different socioeconomic and demographic determinants of health. For example, a possible explanation of increased negative health outcomes in these sub-populations may be decreased access to care, potentially due to financial, mobility, or infrastructural limitations. Given this, mitigation strategies for these sub-populations will be most successful if customized to the needs of each group. For example, males are more likely to be employed in jobs requiring outdoor labor. They may benefit from increased education or being required to take indoor breaks at specified time intervals, while elderly populations who are more sensitive to heat stress, even indoors, may benefit from having air conditioning units installed in their homes.

It is of interest to note that minority status and language did not have a strong relationship with health outcomes, given that supporting literature cites race and/or non-white status as associated with heat-related outcomes [7,13,19,21,22] and many of the areas with increased rates of heat-related health outcomes are in areas where there are high numbers of Hispanic farm laborers and families [33]. However, ethnicity was not reported in the ED visits dataset, thus the percentage Hispanic could not be ascertained, and it is possible that there is a healthy worker effect in these agricultural areas despite race or ethnicity. Additionally, there was no available data to include percentage of non-air-conditioned homes in our analysis, a variable considered predictive of the geography of extreme heat-related illnesses. Despite these limitations, CDC SVI provides important insight into locations where populations may be at increased risk for heat-related illnesses and highlights the importance of utilizing emergency planning tools to prepare for more routine health disasters.

This study had several other limitations. First, CDC SVI incorporates only publicly available census-calculated data and thus does not account for other probable social risk factors, such as occupation or frequency of physical exercise. Second, this study exclusively examines Georgia, a southern U.S. state having a humid, subtropical climate, and which often sustains long periods of hot summer temperatures. It is plausible that many people who choose to live and work in these areas are acclimated to the temperatures and exhibit more heat tolerance than people in more temperate climes, thus while these results are internally valid to Georgia, they may not be valid across regions having typically milder summers. Additional research should be conducted to determine the utility of CDC SVI for heat-related health outcomes in other locations. Third, the database we used includes ICD entries for up to ten underlying causes of death or illness for each patient. If any of these entries contains an ICD code relating to heat for underlying cause of morbidity/mortality, we included that patient. However, there is a small possibility that heat was overlooked as a contributor when multiple symptoms are occurring, and the number of individuals experiencing heat-related health outcomes was underestimated.

Further, misclassification may have been introduced as mortality data were only accessible at the county level. CDC SVI is originally calculated at census tract level, and as such, its rankings may vary significantly across a county. Thus, some individuals included in this study may have been represented by a social vulnerability ranking that was not indicative of their community’s actual social vulnerability. This was more likely to happen in cases where someone’s CDC SVI ranking was more extreme (low or high), which would cause results to trend towards the null. Additionally, as indices combine multiple variables to measure an intrinsically unquantifiable construct, some level of uncertainty is implied. Still, this underscores the importance of completing follow up research related to heat-related health outcomes and social vulnerability at varying temporal and spatial scales and with various measures of social vulnerability to confirm whether consistent associations exist.

Finally, while the spatial and statistical analyses of heat-related hospital ED visits data are complementary, the low numbers in hospital mortality data may have affected the spatial analysis of that dataset. Poisson regression, which is designed to account for small numbers and zeros, was used for the traditional statistical analysis; however, spatial analyses are less effective, with small numbers and zeros leading to unstable mortality rates. Empirical Bayesian smoothing was applied to stabilize the rates; however, this method results in modeled rates for use in the analysis. Although this method is supported in the literature [34], and the spatial analysis results are supported by the statistical analysis, it is possible that the modeled results misrepresent mortality in Georgia counties. Future studies looking at the spatial and statistical relationship of heat-related mortality and social vulnerability over multiple scales and geographies should be completed to strengthen the implications of these results. Additionally, comparing this data to other measures of social vulnerability may further validate these findings.

This analysis evaluates the applied utility of CDC SVI as a standalone tool in identifying counties that are more likely to experience heat-related health outcomes in Georgia and thus may require increased resources and communications during a hazardous heat event. Further research characterizing the predictive value of CDC SVI and demonstrating its role among other known heat-related risk factors may be of value when seeking to understand the inferential capacity of social vulnerability in explaining all cause heat morbidity and mortality. Additionally, further study comparing the performance of CDC SVI to other measures of social and heat vulnerability may be of value.

## 5. Conclusion

By confirming spatial and statistical linkages between heat-related health outcomes and social vulnerability, this study presents evidence supporting the utility of CDC SVI in identifying communities with increased likelihood of heat-related health outcomes in various sub-populations in Georgia. Hot spots of high social vulnerability and heat-related health outcomes are identified, and increased heat-related health outcomes are observed in multiple subgroups, including males, blacks, and the 65-and-older age group, and is likely related to multiple contributing factors. Mitigation strategies should consider multiple factors affecting sub-populations in different regions. Where education might make all the difference with one group, policy or financial aid may be necessary to reduce human vulnerability to heat in other groups in Georgia.

Additionally, because CDC SVI encompasses multiple socioeconomic and demographic factors, it can evaluate a number of sub-populations that may be differentially affected by extreme heat exposure at one time. Although the utility of CDC SVI for preparedness related to heat-hazards may be improved when coupled with other community-specific data, these findings support the use of CDC SVI as a tool to identify communities that may be more susceptible to heat hazards in Georgia. For example, the index could be used to initiate planning and, in conjunction with local knowledge, be an effective tool for designating heat-vulnerable areas, estimating numbers of heat-vulnerable populations such as children and individuals over 65 in those areas, and targeting increased communication, education, public health interventions, and financial resources to those in greatest need, prior to and during extreme heat events. Furthermore, given these findings, in a time when climate change is increasingly at the forefront of our attention, this index offers a relevant visual, supported by evidence which can be used to engage stakeholders, and increase awareness by helping people connect data to place.

## Figures and Tables

**Table 1:** Descriptive statistics for ED visits, mortality, and CDC SVI ranks across counties in GA.

	ED Visits	Mortality	CDC SVI
	N (per 100,000)	N (per 100,000)	Ranking
Min	0	0	0.0
Max	446.8	17.2	1.0
Mean	212.1	1.5	0.6
Standard Deviation	87.9	3.2	0.2
Total in Georgia	14,244	94	N/A

**Table 2:** Social vulnerability index: Themes and variables.

SVI Theme	Variables Included
1. Socioeconomic Status	% Below Poverty Level % Unemployed Per Capita Income
	% Age 25 or Older with No High School Diploma
2. Household Composition & Disability	% Age 65 or Older
	% Age 17 or Younger % Single Parent Household
3. Minority Status & Language	% Minority
	% Age 5 or Older Speak English “Less than Well”
4. Housing & Transportation	% Multi-Unit Structures % Mobile Homes
	% Crowding (More people than rooms) % Households without a Vehicle % In Institutionalized Group Quarters

**Table 3:** Descriptive demographic statistics for a comparison of the breakdown of study groups by race, sex and age.

Race^a^	Georgia		ED Visits		Mortality	
	N	Percentage	N	Percentage	N	Percentage
White	5,556,989	67.54%	8666	60.84%	45	47.87%
Black	2,384,774	28.99%	5163	36.24%	49	52.13%
Asian	181,306	2.20%	59	0.41%	0	0.00%
American Indian	24,509	0.30%	31	0.22%	0	0.00%
Pacific Islander	5392	0.07%	3	0.02%	0	0.00%
Multi-Racial	74,333	0.90%	322	2.26%	0	0.00%
Sex						
Male	4,047,070	49.19%	10,630	74.62%	61	64.89%
Female Age Groups	4,180,233	50.81%	3614	25.37%	33	35.11%
17 and under	2,177,592	26.47%	2431	17.07%	1rowhead4	14.89%
18–39	2,841,678	31.86%	4459	40.59%	16	12.77%
40–64	2,419,593	29.41%	4188	32.97%	30	37.23%
65+	788,440	9.58%	1334	9.36%	34	36.17%

a Racial breakdowns may include both Hispanics and non-Hispanics.

**Table 4:** Results of bivariate global Moran’s I analysis for heat-related ED visits and mortality.

CDC SVI Theme	Overall CDC SVI	CDC SVI Theme 1: Socioeconomic Status	CDC SVI Theme 2: Household Composition and Disability	CDC SVI Theme 3: Minority Status and Language	CDC SVI Theme 4: Housing and Transportation
ED Visits	0.21	0.24	0.21	0.09	0.19
p-value	<0.001***	<0.001***	<0.001***	0.015*	<0.001***
Mortality	0.04	0.08	0.06	−0.02	0.05
p-value	0.11	0.018*	0.055	0.280	0.090
EBS Mortality	0.21	0.24	0.22	0.05	0.21
p-value	<0.001***	<0.001***	<0.001***	0.062	<0.001***

*** p < 0.001

** p < 0.01

* p < 0.05.

**Table 5:** Spatial lag models total heat-related ED visits by CDC SVI theme.

Model	Independent Variable	Est.	Spatial Lag	*R^2^*
Theme 1	Socio-Economic Status	1.899*	0.267*	0.230
Theme 2:	Household Composition and Disability	2.052*	0.292*	0.227
Theme 3:	Minority Status and Language	1.349*	0.401*	0.172
Theme 4:	Housing and Transportation	1.754*	0.305*	0.200
Overall CDC SVI:	All 4 themes	1.927*	0.305*	0.242

* p < 0.05.

**Table 6:** Rate ratios for total heat mortality by CDC SVI theme.

Model	Covariates	Est.	95% CI	P-value
Theme 1:	Socio-Economic Status	1.31	1.18–1.46	<0.01**
Theme 2:	Household Composition and Disability	1.31	1.16–1.47	<0.01**
Theme 3:	Minority Status and Language	0.99	0.90–1.17	0.99
Theme 4:	Housing and Transportation	1.40	1.23–1.59	<0.01**
Overall CDC SVI:	All 4 themes	1.31	1.16–1.47	<0.01**

*** p < 0.001

** p < 0.01

* p < 0.05.
